# A Single-Site Qualitative Study Exploring What Cancer Patients and Health Care Professionals Consider to be the Core Priorities in Relation to Psychosocial Cancer Care

**DOI:** 10.1177/10732748251356320

**Published:** 2025-08-13

**Authors:** Zoe Clothier, Jenny Harris, Agnieszka Kehinde, Victoria Mumford, Kate Upshon, Clare Williamson, Rachel MacArthur, Rachel Stevenson, Sophie Otter, May Teoh, Jo Armes

**Affiliations:** 1 Faculty of Health and Medical Sciences, School of Health Sciences, 105648 University of Surrey, Guildford, UK; 2 Royal Surrey Cancer Centre, 3661 Royal Surrey NHS Foundation Trust, Guildford, UK

**Keywords:** psychosocial, priorities, cancer, patient, health care professionals (HCPs)

## Abstract

**Introduction:**

A cancer diagnosis often impacts emotional wellbeing, with patients frequently reporting unmet psychosocial needs throughout their care pathway. Needs-based support can help address these challenges, but barriers exist that limit its uptake by patients and its provision by health care professionals (HCPs). Identifying key aspects of psychosocial support and tailoring this to meet the preferences and priorities of the individual can enhance the patient-centredness of care, improving both patient outcomes and HCP job satisfaction.

**Aim:**

This study aimed to explore what cancer patients and HCPs consider to be the core priorities in relation to psychosocial cancer care.

**Method:**

Semi-structured interviews were conducted with cancer patients and HCPs at a single UK cancer centre to explore the research question. Interview transcripts were analysed using Framework Analysis to identify and interpret key themes.

**Results:**

A total of 21 interviews (10 with patients, 11 with HCPs) were analysed. Six cross-cutting themes were generated from the data: *personalised support, awareness and accessibility of support, patient-HCP communication, coordination of care, time and resource constraints,* and *‘what matters most’ in psychosocial cancer care*. HCP-specific themes were *recognition of HCPs’ own psychosocial needs* and *the necessity for education and training in psychosocial care.*

**Conclusion:**

Psychosocial care is pivotal to cancer patients’ wellbeing and positively influences their perceptions of health, especially when this aligns with their individual needs and preferences. Cross-cutting themes highlighted the importance of holistic approaches addressing the multifaceted needs of patients and indicated alignment between patient and HCP care priorities. Supporting HCPs’ own wellbeing and providing appropriate training and skill development may further enhance the quality of psychosocial care delivery. Implementing a stepped-care approach may enable practitioners to better support their patients which in turn will improve their job satisfaction and patient outcomes.

## Introduction

A cancer diagnosis can be distressing and presents an increased risk for anxiety and depression.^1,2^ Patients frequently report unmet needs throughout the cancer care pathway^3-5^; notably, unmet psychosocial needs affect up to 69% at diagnosis, 89% during treatment and 17% of long-term cancer survivors^6^ illustrating the spread and extent of these unmet needs. Psychological factors such as anxiety and depression are also associated with reduced quality of life, impaired social relationships and longer time to adjust in cancer patients.^7,8^

A study conducted across four Italian hospitals found that an estimated 40% of cancer patients experience a psychological condition which reaches a diagnoseable threshold^9^; in a review by Kuhnt et al^10^ the 12-month prevalence of anxiety disorders was 15.8% and for mood disorders this was 12.5%.^10^ One study of 55 North American cancer centres found that 2157 (46%) patients experienced significant psychosocial distress,^11^ and another study of 1966 Australian cancer survivors found that 32-44% reported distress over a 5-year period.^12^ These statistics reinforce that cancer survivors may still require support following the end of treatment, a commonly noted observation in the literature.^13,14^ Taken together, this highlights the necessity of psychological support for people living with cancer throughout and beyond the cancer care journey.

Psychosocial support is important for patient wellbeing,^15,16^ serving as a key determinant of quality of life.^17^ It encompasses emotional and information needs, effective communication with health care professionals (HCPs) and practical support.^18,19^ When unmet, psychosocial needs are associated with poorer quality of life for patients.^20^ A recent systematic review of qualitative research outlined the impact of patients’ unmet psychosocial needs as including poor survivorship planning, feelings of abandonment and financial and employment issues,^21^ illustrating the range of areas impacted when psychosocial support is not offered to the extent desired by the patient or in line with their unique care needs. Therefore, psychosocial needs should be addressed as a priority throughout all stages of the pathway.

Cancer care guidelines increasingly emphasise the importance of addressing psychosocial wellbeing. Internationally, cancer policy highlights the value of offering personalised care including tailored information and support for patients.^22,23^ Similarly, the International Psycho-Oncology Society recommends implementing psychosocial care into routine practice.^24^ Guidelines for optimal cancer care provision, including psychosocial care, have been produced by the European Society for Medical Oncology (ESMO),^25^ National Health and Medical Research Council (NHMRC) in Australia,^26^ the Canadian Association of Psychosocial Oncology (CAPO)^27^ and American Society of Clinical Oncology (ASCO).^28^ International guidance has also been developed by the Multinational Association of Supportive Cancer Care (MASCC),^29^ indicating thorough recognition of psychosocial cancer support as integral to patient care.

Several barriers impede the provision and uptake of psychosocial support, however. Patients may decline psychosocial support due to perceiving it as unnecessary^30^ or face practical challenges with access such as travel difficulties.^31,32^ For HCPs, barriers to providing psychosocial care include communication challenges,^33,34^ heavy workloads, time constraints^35^ and viewing psychosocial care as beyond their role.^36^ Many HCPs feel insufficiently trained to deliver psychosocial care and highlight the need for additional education.^35,37^ Illustrating this inadequacy, a recent qualitative study of allied health professionals (AHPs) found only 25% were aware of the UK National Institute of Care Excellence (NICE) cancer care guidelines regarding psychosocial support, and 54% reported having received psychological skills training.^38^ Similarly, research by ESMO found that HCPs report a lack of knowledge and can experience uncertainty when providing psychosocial care, particularly to adolescents and young people with cancer.^39^

Importantly, HCPs themselves may also have unmet psychosocial needs, which can negatively affect patient satisfaction^40^ and contribute to workplace stress and professional burnout.^41^ Compassion fatigue is another well-documented experience among oncology HCPs^42^ and describes cumulative distress and exhaustion from caring for others.^43^ Compassion fatigue contributes towards physical and emotional exhaustion, which can reduce job satisfaction and ability to perform their job correctly.^44^ Similarly, cancer HCPs can experience moral distress, which describes emotional discomfort when care provision does not align with their own perception of what entails high-quality care.^45,46^ In this way, moral distress can further contribute towards burnout and detrimentally impact clinician wellbeing.^47,48^ As such, it is important to be mindful of HCPs’ wellbeing to ensure high-quality patient care and a resilient workforce.

Patient-centred care (PCC) refers to treatment being tailored to the unique needs of the patient, giving them a central role in their own care^49^ and enables healthcare practices to become more aligned with what patients want by identifying and addressing the aspects of care they consider as being most important.^50^ It actively involves patients in decision making, emphasises their relationship with HCPs^51,52^ and is a key indicator for care quality.^53^ PCC also facilitates optimal care practices by enabling HCPs to better understand and address individual patient priorities,^50,54^ helping to ensure specific patient care needs and preferences are addressed. Understanding patients’ and HCPs’ priorities in psychosocial cancer care is essential to achieving a more patient-centred approach, as well as improving HCPs’ job satisfaction and quality of care.

This qualitative study aimed to explore key aspects of psychosocial cancer care that were identified as core priorities for cancer patients and oncology HCPs; these priorities are presented as themes in this study. ‘Themes’ broadly represent perceptions, experiences, values and emotions that cannot be directly observed. Rather, they are recurring patterns identified by the researcher during data analysis, which are grouped to form a representative thematic framework^55^ from which meaning can be derived.^55^

## Method

### Design and Context

A qualitative design using semi-structured interviews was used to explore psychosocial cancer care experiences. The reporting of this study conforms to the Consolidated criteria for Reporting Qualitative Research (COREQ) guidelines.^56^ Participants included cancer patients and oncology HCPs (including consultants, registered nurses and allied health professionals (AHPs, including radiographers, dietitians and acute oncology trainees)) who were recruited from a single UK cancer centre (June-August 2023).

### Sample and Recruitment

We aimed to recruit a minimum of 10 patients and 10 professionals to ensure we achieved sufficient information power.^57^

#### Patients

Eligible patients were purposively recruited by a member of the clinical team (AK), who briefly explained the project via email or over the telephone and invited patients to participate in an interview if they met inclusion criteria (below). Consent to share their contact information with the interviewing researcher (ZC) was obtained. ZC provided each patient with an information sheet explaining the study in full. Interviews were then arranged with consenting patients. To participate, patients needed to have a diagnosis of primary skin, lung, brain or prostate cancer, be aged 18 or older, cognitively and emotionally capable of engaging in an interview and able to provide informed consent. Patients received a £20 voucher as reimbursement for their time and contribution to the study.

#### HCPs

A recruitment email outlining the project was sent to purposively selected cancer HCPs by a member of the clinical team (AK). Email addresses of interested HCPs were then shared with the interviewing researcher (ZC) with consent, who provided information sheets and arranged the interviews. To participate, HCPs needed to be employed by the hospital and work with cancer patients receiving treatment for their condition.

### Interview Procedure

Interviews were arranged by the researcher for a mutually convenient time and were conducted one-to-one with the interviewing researcher (ZC) and the participant. Potential participants were provided with an information sheet and given an opportunity to ask questions at least 48 hours before the interview took place. Interviews took place either over the telephone or via MS Teams and participants were given the option whether these were recorded with video or audio only. Verbal consent to participate was recorded prior to the interview, and this recording stored separately from other research activities. With consent, interviews were recorded and subsequently transcribed using the in-built MS Teams transcription feature and checked for accuracy by the interviewing researcher (ZC). No repeat interviews were conducted. One patient requested to be sent their transcript for review prior to data analysis but confirmed they had no comments on its content; no other transcripts were returned to participants.

To keep interview content focused topic guides were developed. Development of the topic guides was informed by a rapid review of psychosocial cancer patient reported experience measures (PREMs) conducted by the authors^58^ and further refined with input from all authors. Topic guide content included experiences of either providing (HCPs) or receiving (patients) emotional support, other forms of support that were important for patients’ wellbeing, notable challenges related to the cancer journey and what were perceived to be the core priorities for patients and HCPs in relation to cancer care. Responses were probed for further detail as needed. Topic guides and prompts for the questions can be found in Supplement 1.

### Analysis

Interview transcripts were anonymised prior to analysis by removing any identifying information and redacting names and location information when mentioned. Data were analysed using Framework Analysis^59^ in NVivo12 software; this method is particularly useful for applied research.^59^ The aim of Framework Analysis is to identify, describe and interpret overarching patterns within and across individuals and themes related to the research topic.^60^ Framework analysis comprises five key steps in the analytic process: data familiarisation; framework identification; indexing; charting; mapping and interpretation.^59^ An inductive analytic approach was adopted, allowing themes to be generated directly from the raw data without reference to any prior theory and to preserve participants’ voices without imposing the researchers’ own views on these.

Following framework analysis guidance, one author (ZC) initially familiarised themselves with the data and developed the preliminary thematic framework focused on moments of interaction and support. The thematic framework was developed using a recurring process whereby themes were refined and revised repeatedly to ensure they accurately represented the data, and codes were developed throughout the analytic process. A second author (JA) independently coded a subsample (n = 4, 19%) of the transcripts to further refine the framework and ensure it captured meaningful, challenging or impactful moments of the cancer journey. Authors (ZC, JA) met once a month throughout the data analysis process (August-October 2023) to discuss code definitions, relevance and to make sure codes were not interchangeable or redundant. Transcripts were coded systematically, and these codes were charted into themes that captured the most salient aspects of psychosocial care, allowing direct comparisons both within and between participant groups (patient or HCP) to be made. This later process aided meaningful interpretation of the themes identified.

### Reflexivity

In line with the Consolidated criteria for Reporting Qualitative Research (COREQ)^56^ guidance, it is important that authors are transparent about and consider the potential influence of their personal characteristics and preconceptions on data collection and interpretation. The interviewing researcher (ZC) is a university-based research assistant who is white, female and has an MSc in Health Psychology; participants’ knowledge of this may have biased their responses based on assumptions regarding the interests and expertise of the researcher, such that answers were more socially desirable. Gender dynamics could have played a role in how participants engaged with the interview questions, particularly in discussions of sensitive or intimate topics. Similarly, differences in ethnicity or education level may have affected participants’ comfort in sharing certain perspectives. Hence, these factors could have contributed to participants’ responses especially where dissimilarities arose. The interviewing researcher is also neither an oncology HCP nor has lived experience of cancer, which was borne in mind throughout the research process for its potential influence on rapport with participants and her own understanding of discussion around medical issues and treatments. To overcome this, co-authors with expertise in oncology research (JH, JA) were consulted where uncertainties arose.

The interviewing researcher is not employed by the cancer centre and had no prior relationship with participants, which potentially facilitated a more open discussion; patients and HCPs may have felt they could speak without concern about participation negatively impacting their treatment or employment respectively. The researcher was also aware that interactions and interview dynamics with HCPs may have been affected by her lack of medical training and background.

### Ethical Considerations

This study was reviewed by an independent Research Ethics Committee and was granted a Favourable Ethical Opinion by East of England - Cambridgeshire and Hertfordshire Research Ethics Committee on 03/04/2023 (REC Ref Number: 23/EE/0057).

## Results

### Sample

A total of 21 interviews were conducted. Sixteen patients consented to be contacted and 10 (4 female) participated (62.5%); those who did not participate either did not respond to the interview invitation (n = 5) or were too busy to take part (n = 1). The median duration of patient interviews was 39 min (range 27-70 min). Four patients had a primary diagnosis of melanoma, two had prostate cancer, two had lung cancer and two had brain cancer.

Eleven HCPs (9 female) expressed an interest in the research, all of whom participated. This consisted of five Allied Health Professionals (AHPs), three Registered Nurses (RNs) and three clinical oncologists. The median duration of HCP interviews was 29 min (range 13-39 min) (Tables 1a and 1b for details).

**Table 1a.** Patient Characteristics

ID	Sex	Primary diagnosis
P1	F	Lung
P2	M	Melanoma
P3	M	Prostate
P4	F	Brain
P5	M	Prostate
P6	M	Brain
P7	M	Melanoma
P8	M	Melanoma
P9	F	Lung
P10	F	Melanoma

**Table 1b.** HCP Characteristics

ID	Sex	Professional group^a^	Approx. time in current role
H1	M	AHP	5 years
H2	F	AHP	13 years
H3	F	RN	5 years
H4	F	AHP	5 years
H5	M	AHP	9 months
H6	F	CO	5 years
H7	F	RN	1 year
H8	F	CO	9 years
H9	F	RN	6 years
H10	F	AHP	3 years
H11	F	CO	15 years

^a^AHP, allied health professional; CO, clinical oncologist; RN, registered nurse.

### Themes

A total of eight themes were identified from the data, six of which were present in both patient and HCP accounts although in different ways. These cross-cutting themes were *personalised support*, *awareness and accessibility of support*, *patient-HCP communication*, *coordination of care*, *time and resource constraints* and *‘what matters most’ in psychosocial cancer care*. Additionally, there were two further themes that were unique to the HCP interviews: *the necessity for education and training* and *recognition of HCPs’ own psychosocial needs*. After completing data collection and analysis, themes were presented back to patients and HCP in focus groups to obtain their feedback about the proposed themes.

**Figure 1.**
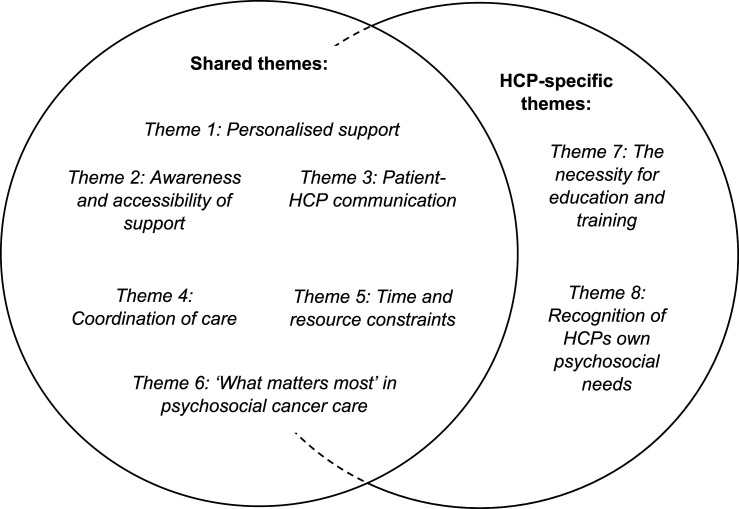
Thematic Map Illustrating the Main Themes and Overlap of These Between Patients and HCPs

See Figure 1 for a diagram illustrating the themes and overlap between patients and HCPs.

Themes will each be discussed below; full definitions of themes are presented in Table 2 (see Supplement Table 1 for themes and illustrative quotes). Where quotations are presented, HCPs are denoted with an ‘H’ and patients with a ‘P’.

**Table 2.** Themes and Definitions

Theme	Theme description
Personalised support	Providing psychosocial care that is tailored to each patients’ own needs; the role of individual differences (including personality, long-standing coping styles and support preferences)
Awareness and accessibility of support	Patients know what additional support and resources are available to them, and HCPs are aware of these resources to signpost patients towards
Patient-HCP communication	Interactions between patients and HCPs, and its importance for patient wellbeing
Coordination of care	Patients’ and HCPs perceptions of care being coordinated
Time and resources constraints	Views around time and resources as being a barrier to receiving or providing psychosocial cancer care
‘What matters most’ in psychosocial cancer care	What patients and HCPs identify as being important in providing or receiving psychosocial cancer care
The necessity for education and training in psychosocial care	HCPs views around the importance of education and training in delivering high-quality psychosocial cancer care
HCPs own needs in providing psychosocial care	What HCPs consider important for themselves in relation to providing psychosocial cancer care

### Personalised Support

Personalised support whereby care is tailored to meet the individual needs and preferences of patients was frequently mentioned by patients, with one commenting “*it’s quite nice that it’s very personal*” *(P8)*. Another explained that the tailored care they received from all HCPs at the hospital was appropriate for their needs and contributed towards their sense of *“feel(ing) cared for” (P9)*:

They are very caring… they support you as much as, you know, as you need. And if you have questions, they will always try and get the answers and things like that. – P9

The nurses were seen as approachable by patients, which contributed towards their sense of being supported:

The lovely nurses at the hospital have been fantastic for me to talk to and chat to, and funnily enough, not even about illness but about other things too. Yeah, great team, very supportive. – P7

One patient described how they perceived personalised support as encompassing emotional aspects as well as providing information about their condition, explaining how the nurses adapted the care they provided to meet their individual needs:

I think maybe that’s because that is part of their role, to support not only with knowledge and what’s happening, but also to support the person as well. – P4

There was, however, acknowledgement that emotional support was not unanimously needed, with some patients preferring informational support. One patient explained:

I’m the sort of person that prefers to actually know what’s happening, and what’s going on. But as I said, everybody’s journey is different and it’s a very unknown situation. – P9

For some patients, personalised support did not necessarily entail emotional support, illustrating the diverse range of ways in which patients can feel cared for. Demonstrating this, one patient described how practical aspects of their care were important as this enabled them to continue working:

As part of (my job), we have a limited exposure in millisieverts per year, which the CT scans for my immunotherapy would be giving me, and the team said I can have full-body scans instead… which is absolutely brilliant. – P8

Other patients expressed that they *“haven’t needed that kind of (emotional) support” (P3)* or described themselves as *“a person who likes facts and figures” (P9),* highlighting the different ways in which support can be tailored or differently required. In line with this, an oncologist acknowledged that *“some people feel (emotional support)’s not their kind of thing” (H6)* which meant that resources were not necessarily required or utilised by all patients.

Providing personalised care specific to the needs of the patient was of clear importance to HCPs. One AHP explained that they wanted to ensure patients were *“seen not as a disease but as a person” (H10)*, with a nurse further explaining:

It’s about putting your bias to one side, because that’s what we do as professionals, and treating everyone as an individual, which we need to do because not everybody’s pathways are the same, despite their disease being the same. – H7

As with the patients, HCPs acknowledged there was *“huge variation” (H6)* in patients’ needs. There was an appreciation regarding the importance of recognising the diversity of support requirements and matching care provision to these:

So yeah, it’s trying to work out what it is, you know, their particular need is and how they’re going to find the support for that need. – H4

Assessing what patients’ unique needs are and providing support appropriate to meet these needs was identified as vital by nurses and AHPs, who recognised that each patient has different priorities for their care*:*

What is important to one person is not to the other, and it’s not about generalising and putting people into boxes and categories. It’s about looking at the individuals and what they need. – H7

In contrast to the nursing team, oncologists did not consider providing emotional support to be an aspect of their “*clinical” (H8)* role, with one explaining:

My skills would not be used well if I was offering psychosocial support myself, my skills and speciality over the years has been to treat cancer. – H11.

Another explained that they *“would defer to (their) CNS’ [clinical nurse specialists]” (H8)* for emotional support provision, with a third contrasting their role with the *“softer approach” (H6)* of the nurses:

The CNS’ help to bridge the gap between the medical team… and the softer approach of being there, allowing the patient to have some time to talk about what’s really bothering them. – H6

The format in which support is presented was also acknowledged as affecting whether a patient utilises this or not, indicating the need to have resources available in different formats so that it reaches the broad range of patients who may need it:

There are some patients who devour a written leaflet and others who just leave it in the folder, but if you give them a phone with a video on it, then that’s really helpful. So I think whatever support is offered, it needs to be in different formats so that you’re accessing the information to help. – H2

### Awareness and Accessibility of Support

Patients generally felt well-informed about the additional resources available to them. Referring to the information and support centre based at the hospital, one patient noted:

I was made aware that it was available to me. I was told the things that they offer and if there’s anything that I wanted then I could call them and make an appointment. – P1

HCPs also mentioned the information and support centre, describing it as a *“fantastic” (H3)* resource. They felt it was *“a relatively straight forward thing” (H1)* to direct patients towards given its proximity to the cancer centre itself.

Patients identified various barriers to accessing resources and support however, including practical challenges such as *“the logistics of getting to the cancer centre” (P8)*. Another patient acknowledged how these barriers would prevent them using the information and support centre despite the potential value of such resources:

I’ve had the opportunity to go back there if I ever want to, and I’m very grateful, but it’s not a very local hospital to me. – P7

One patient noted that to benefit from some of the services available, this would involve disruption to their workday which prevented them from accessing support:

Those kind of support groups are great, but if it’s, you know, support groups who only meet on a Friday at 10 a.m., it’s quite tough. There’s a huge amount of people with cancer who work in the day. – P2

Not all patients were aware of such additional resources, however, with one commenting that they *“didn’t know what was available and… what to ask for” (P6)*.

HCP accounts, echoing the patient experiences, recognised the value of patients being able to access additional resources to support their wellbeing:

I think having this option of being able to access support, whether it is in the form of counselling or any other form, is really important. But you know, not all the NHS places have that. – H11

HCPs also reflected on the self-referral process required to access some resources as a prohibiting factor, describing it as a *“difficult… first step” (H6)* for patients to initiate the process of accessing psychosocial care. One nurse explained how they thought the self-referral process might impede the uptake of support:

(Patients) have to self-refer, which is quite difficult… I think that’s really hard, if you’re in a really low place to, even though it’s online, to just put your details in and to refer yourself. – H3

The impact of the self-referral process to receive additional support also featured in patients’ discourse, with one patient reflecting on their experience of this:

If it had been like, someone from the (information and support centre) is gonna call you and they’re gonna sort you out some free treatment, that would have been amazing. – P2

HCPs also reflected on the importance of their own awareness of available services to better refer patients appropriately, with one oncologist commenting on how this interacted with their ability to signpost patients:

I’m very keen for us to have a better understanding and knowledge of what is available… I think a lot of people are not aware of what we have available here. – H6

Additionally, HCPs acknowledged the range of barriers that may contribute to a *“disparity of the psychosocial elements” (H6)* available to patients. One AHP elaborated on how patients’ own beliefs around their diagnosis could interplay with this:

Lots of reasons, whether it be mobility or stigma… prevents people accessing services because they almost feel like they deserve their diagnosis. So, bringing the service to the department would hopefully encourage those people to go and engage with it. – H5

Discussion also included how, as an HCP, the process of signposting patients to additional resources can be an obstacle when referral pathways are not clear:

I don’t think there’s enough joined up writing as far as signposting people, and I think it would support us to be able to say, ‘okay, so this patient is struggling with this, this is a resource they can access’… then you can point them in that direction. – H2

Similarly, the issue of whether current support services align with patients’ preferred means of receiving support featured in one nurse’s account, who considered whether these were equitably accessible to all patients who may need them:

The majority of this information are communicated via Internet, Instagram, even ALK [anaplastic lymphoma kinase] patient support groups started getting a TikTok, so they’re all online. But for older generations, they may not use that. – H9

### Patient-HCP Communication

Patient-HCP communication emerged as an important determinant of patient care across all accounts. Patients particularly valued when HCPs interacted with them in a *“gentle” (P9)* and appropriate manner. One patient shared their positive experience with the cancer care team:

They will talk to you properly. They all, there’s no condescending, there’s no patronising… they’re fantastic. – P6

Patients acknowledged the positive psychological benefits of being recognised by HCPs during hospital visits. This personalised approach was seen as important for the therapeutic relationship with the cancer care team and for their wellbeing:

You’re not just a name and a hospital number. You’re an actual person, and you can tell they genuinely, you know, they do care. You build relationships with them and that helps as well. – P9.

HCPs were similarly aware of the importance of communication, identifying how *“the language (we) use” (H3)* directly affects patients’ wellbeing. One AHP noted how this interaction influenced how they discussed patients’ condition with them:

What we do well is… explain to patients if their disease is curable. We are aiming to cure them… and I think that kind of alleviates their anxiety a bit. So using words like ‘curable’ [and] ‘curative’, I think those words really help. – H1

Non-verbal communication was also acknowledged as having a key role in patient communication, with HCPs recognising this. These *“little things… such as your body language, your tone” (H1)* were considered integral. It was recognised that due to an increased number of appointments taking place virtually, HCPs could *“miss those subtle signs…that someone isn’t doing as well” (H6)*, however.

One patient noted how the mode of communication affected their experience of care delivery, explaining that it would be *“nicer to see people than to have phone appointments” (P5)*. HCPs agreed that the method of consultation affected psychosocial care delivery, commenting that *“you pick up so much more” (H4)* when assessing patients face-to-face. One nurse elucidated this difference, contrasting their experience of online vs in-person appointments:

I think if you review a patient face-to-face, or review a patient on the phone, you can get a different kind of impression about what psychosocial needs they have. – H9.

A clear contrast between online and in-person consultations was also made by an oncologist, who explained their preference:

I much prefer to see people face to face, especially if I am delivering bad news. I don’t want to be doing that over the telephone, I want to be doing that in a more personable way sat in front of the patient, with a CNS ideally. – H6.

There were also areas for improvement identified. Some patients expressed a preference for information in more accessible formats to help them understand what the medical team had told them during their consultations. One patient explained:

If I was being pedantic about it, I would like that information written down. The information (the doctors) gave me was very, very useful, but I didn’t have it written down. I did have a cancer care pack given to me, but it was more generic. It didn’t have the information specific to my care written. By the time I got home I’d forgotten names, timings, issues, all of that. – P8.

Additionally, some patients noted that they had not directly met their consultant to discuss their health status and care plan. One patient felt such a consultation would be psychologically beneficial and *“massively reassuring” (P3)*:

The fact that I’ve never met my consultant is a negative. I’d have liked to have met them. I would still like to meet them at some stage… I don’t know whether I’ve missed anything or not, by not meeting the consultant. – P3

Another patient explicitly expressed their preference for being able to speak to their consultant compared to other members of the medical team, explaining that being reviewed by their consultant contributed to their sense of consistency in the care they receive:

If I see the consultant, that’s fine. The continuity of care is there. But on the other hand, if you see another doctor or something, they might have briefly read your notes, but they don’t really know you, or what your journey has been, or what’s happening” – P9

In contrast to this, one patient mentioned that although they had *“never met [the consultant]” (P4)*, this had not affected them to the same extent due to their positive relationship with their nurse. They continued:

…but you know, that’s fine. (CNS) would be my go-to person, she’d be there for me if I’ve got a concern. – P4.

Additionally, HCPs recognised the central role of communication in patient satisfaction, as detailed by one oncologist:

A lot of the anxieties that patients have, all the complaints that we end up receiving, are almost always down to… some kind of miscommunication. – H6

Seeming to reflect this sentiment, one patient provided an example of a negative interaction at the hospital, although this was identified as happening only on *“one occasion” (P3)*:

There was one occasion early on when one of the reception staff, I thought, was extremely rude. I reported that, and one of the nurses came and saw me… There’s no excuse. I never saw that receptionist again, but I mean, just train her, you know? – P3.

In contrast, another patient reported positive experiences *“from the receptionist right the way through” (P10)* with the wider healthcare team:

I’ve been very fortunate… I mean, it’s wonderful, I could go to anybody to speak to them about anything. They’re all very kind. – P10.

### Coordination of Care

Patients had differing experiences of coordination of care, with some describing it as *“like a well-oiled machine” (P10)* and *“all really smooth” (P1)*. Collaboration within the nursing team was viewed as important, with the nurses considered by one patient as *“the linchpins of it all” (P2)*. Having well-organised care clearly benefitted patients and supported their sense of wellbeing:

When I go for treatment, they’re all very good. They all know what’s happening, if medication has been prescribed for me from the doctor, that’s there for me, always. There’s never been any hitch in anything, it’s amazing. – P10

Other patients described contrasting experiences, however, with this identified by some as the *“downside” (P5)* of care provision from the cancer centre. One patient described the disjointed care they received between different departments of the hospital:

I mean, each department treats you fairly well when you’re there, but there doesn’t seem to be too much coordination between them. That’s where it falls down really. There should be some connection. – P5

Another patient similarly expressed that care was not always cohesive, drawing on their experience of trying to receive scan results from a previous appointment:

When I actually went in to get my results, they didn’t have all of them, so I had to wait a further two weeks... we’d been going over there to get the results, so it was a bit like, ‘oh, I’ve come away without the answer’, really. – P4

While experiences of internal hospital or multidisciplinary team coordination were mixed but generally positive, cross-site communication between different geographical locations or organisations was seen as more fragmented and uncoordinated. One patient commented, *“different Trusts [organisational unit within England’s NHS], communication is never great” (P8)* with another referring to their experience of coordinating appointments at their local general practice alongside hospital appointments:

‘Challenging’s’ the wrong word, but I have to sort of keep an eye on it… I’ve got my review coming up and I have the challenge of making sure that I’ve booked in for blood test at my local doctor’s… My doctor’s is ‘under special measures’. – P4

HCPs also acknowledged the challenges associated with care coordination, noting that patients often face difficulties when their care moved between hospitals. A nurse reflected on this:

It can be (a challenge), when they’re used to one hospital and then they come here and they have to navigate this hospital. As much as you try and do a unified support system, all hospitals work differently. We haven’t got the same databases. – H7.

Patients described how the level of coordination experienced directly impacted timeliness of their treatment and in turn their psychological wellbeing, with one expressing that they were *“very grateful for the speed of it all” (P7)*. However, there was a clear negative impact when there were lengthy intervals between different stages of care:

Most of the time it’s someone saying, ‘I can see on the system you’re meant to have had it, let me chase so and so’… All those things do stress you. Like, the whole thing around scans is stressful, you’re incredibly stressed about the fact that, what’s the news gonna be? Are the tumours still shrinking? Have they come back? So, anything that compounds that does lead to more stress, you know? – P2

The view that prolonged waiting times between appointments detrimentally affected patients was echoed by an oncologist:

What is frustrating, and I think actually does add to the psychological burden for patients, is waiting for results. Well, waiting for a test first of all, then waiting for results. For a patient, you know, you’ve been told ‘you’ve got cancer’, then you’ve got to wait for more scans, then you’ve got to wait for the results… I find that does affect them, psychologically. – H8

### Time and Resource Constraints

Time and resource constraints were identified as two key barriers for HCPs in providing adequate support, with time considered *“probably number one” (H4)* in terms of the challenges HCPs faced when providing psychosocial care. HCPs felt that lack of time impeded psychosocial care delivery, either directly or by limiting their ability to signpost patients to other resources. One nurse commented that *“in the 20-minute consultation there’s not much support (they) can give” (H3),* with an AHP building on this:

Well, the biggest challenge is just time… We try and signpost them to the correct places, but we ourselves just don’t have the time to do it, so all we’re really able to do is signpost people. So the biggest issue is definitely time. – H5.

Although patients were less vocal about these challenges some did recognise the time constraints affecting the health care team, commenting that *“everybody’s up to their eyes in things” (P4)*. Another patient noted:

I think it’s just a case of, you know, they’re all so busy and they have loads of people to see. They just don’t have the time. – P9

There was also reflection on how the different roles of the care team interacted with the depth and nature of information patients disclosed. One AHP identified this, suggesting that having more time to spend with patients facilitated more open conversation:

Our clinics are not governed by 15- or 30-minute slots. I can spend an hour with the patient, whereas the consultant will only have 10 minutes, so I can guarantee the patient’s going to tell me a lot, lot more. They’ll tell me stuff that’s going on that they should have told the consultant but didn’t get the opportunity to. – H2

Similarly, a patient contrasted the relationship they had built with their nurse and another member of the wider healthcare team, attributing this difference to perceived time constraints:

I don’t know what you’d call them, technicians? Not really nurses, but she can take the bloods… I’m probably talking to her longer than I am with (CNS) because, well obviously (CNS) is very busy. – P5.

Despite these time pressures, from the patient perspective, this did not seem to decrease the quality of care they received with many expressing how *“grateful for the team” (P7)* they were. One provided their personal experience throughout their treatment at the cancer centre:

I never felt like (the nurses) were looking at me going, ‘I’ve gotta get on to the next thing’. But you know that they do. You know that they’ve got five other appointments before lunchtime… they’re all run ragged, and they never show it. They all do what they do and smile and carry on. – P2

All HCP groups discussed the difficulty of supplementing care with additional resources, with an oncologist acknowledging that they *“have no capacity to be involved” (H11)* with additional care provision. An AHP further commented that this would “*just require far more staffing” (H5)*, reflecting the challenges they faced when providing additional support to their patients:

It’s all well and great trying to provide the extra psychosocial care, but we are currently stretched with our resources as it is, so there is very little more that we would be able to offer without creating more roles and more posts or asking our support networks to do that instead. – H5

When resources are available, the ease of access to these is important for it to be utilised successfully by patients and HCPs alike, otherwise this becomes another barrier to their uptake:

I also think that it needs to be easily accessible. There is nothing worse than saying, ‘go to this website’ and then when you open the website you just think, ‘hang on a minute, where is it?’. It drives me mad. – H2

A nurse also identified that awareness of resources was key to being able to make onward referrals for patients, explaining that “*if we’re not aware of this, we’re not able to guide the patient to this” (H9)*. Aligning with this view, one AHP explained how they believed additional work outside of their typical role was necessary to know about additional resources available to patients:

I’m not aware of any (additional resources) to be honest, or at least they are not available to me. I think this requires, you know, personal research into this. – H1

Some HCPs suggested that they were currently underutilising existing services and resources for psychosocial support. They wondered whether they could *“be a bit more clever” (H8)* with how they optimise allocation of these resources, suggesting that a needs-based approach might be more efficient:

We maybe need to have a better way of triaging, and you know, sort of allocating the support and the workforce to the various levels of needs that patients have. – H8

Patients also acknowledged these difficulties, recognising that there are *“only so many resources available” (P2)* for them to engage with. One patient described their experience of using the information and support centre based at the hospital, illustrating how use of the centre can be complicated due to its popularity compounded by its limited availability:

I’m sure it provides a lot of relief to a lot of people, and it’s nice knowing that it’s there. But it’s quite hard to get an appointment, as they’re very busy. There’s a lot of people using the facilities. – P1.

However, as with time constraints, patients mostly did not seem to feel resource constraints detrimentally affected their care:

I can only say the medical team have been brilliant. I’ve had all the resources I’ve needed. I’ve had all the care that I needed... I was offered alternative therapies to relax through, also a contact number if I needed to speak to anybody. – P4

### ‘What Matters Most’ in Psychosocial Cancer Care

The importance placed on both physical and psychological wellbeing consistently emerged for patients and HCPs, summarised by one patient as *“an equal, two-prong thing” (P2)*. One HCP identified how physical and psychological wellbeing can be interrelated:

So, I think appearance and mobility… I know that’s a physical thing, but it’s actually the effect of the physical stuff, and the weakness and the frailty, on their psychological wellbeing. – H2

One oncologist suggested that whilst psychological and physical health are both of importance to patients, they believed that patients prioritised physical health above psychological wellbeing:

Survival. The studies generally show that survival is what they value most, but I’m sure they can’t deal without psychological support. So that, that also matters. There’s no doubt about it. – H11

One patient indeed prioritised their physical health and recovery from cancer more so than receiving emotional support, which they felt they *“didn’t need” (P3)*:

Feeling that I am fully recovered, that I’m not gonna be popping my clogs in the near future, which is what it looked like when I was first diagnosed. – P3

Emotional aspects of care were more explicitly valued by other patients, with one considering this as a key factor in their journey through treatment and recovery:

I genuinely don’t, I think, if I didn’t have access to that (emotional) support stuff, it would have been a very different journey for me. – P2

Others highlighted the significance they placed on practical support and *“the flexibility of the teams” (P8)* in accommodating them during their treatment, meaning they had minimal disruption to their usual activities. Another recounted the importance of being able to return to their routine pre-diagnosis:

I’m quite a self-aware person, so for me (emotional support) wasn’t so major… For me, getting back to work was important, as you know, you’re not thinking about your diagnosis, you’re just getting on with your job. – P4

For some HCPs, being able to provide more concrete support through having greater awareness of the available resources to signpost patients towards was a core priority so patients could receive the support they needed, when they needed it:

(It’s important) that I’m able to, you know, provide the support or find where they can find the support elsewhere. I think that’s probably it, either knowing I can offer that support or guidance or where, you know, where I can signpost them to. – H4

In relation to signposting patients one nurse explained their prioritisation of education around assessment tools, which they considered essential in accurately understanding patients’ needs and providing appropriate support for these:

You won’t be able to make a decision, what kind of referral for these patients and to what services, without assessing them. So I think education about assessment tools is very important before signposting anybody. – H9

Both patients and HCPs described how a range of experiences could shape and enhance psychosocial wellbeing, which were identified as being *“a very individual thing” (P9)*. When asked their view of what patients would consider most important, one AHP described a person-centred perspective that considered the patient outside their diagnosis:

(Patients want to) feel listened to, feel they are seen not as a disease but as a person that not only has that condition, but has a family, a life before cancer and a professional background. – H10

Another similarly described the comprehensive care they sought to provide for patients, again viewing them beyond *“just…their disease” (H1)* and incorporating other aspects of wellbeing into their care plan:

I want to feel that I’m giving my patients a holistic management. I’m not just treating their disease, I’m not just treating their side effects, but I’m also capable of, well, if it’s not me personally, but I can provide a holistic management strategy for them that will include their psychological wellbeing. – H1

HCPs also considered how patients’ priorities may change throughout their cancer journey, reflecting the need to adapt care to their evolving individual needs. One AHP summarised:

I imagine that for most patients it depends on where in the journey you’re catching them. If they’re in sort of the survivorship part of their journey, then that’s going to be their primary goal… whereas if you catch them at the earlier stage, they might just want more information about what it is exactly that’s going to be happening. – H5

### Necessity for Education and Training

The necessity for education and training featured prominently as another important factor that is required to develop HCPs’ ability to provide adequate psychosocial support to patients. One nurse shared their opinion that *“psychological training should be embedded into nursing practice” (H7)*. However, it was frequently mentioned that this is not the reality with many commenting on the *“lack of training about delivering psychosocial support” (H1)*. An oncologist noted:

I don’t think that’s something that we really get proper training for at all, actually. So all of us have gone on a communications, an advanced communication skills course. But… it’s not really about providing psychological support for our patients. So actually, there’s a huge hole within our education… we don’t really have that form of (psychosocial) training. – H6

Communication training was specifically identified as an area where HCPs felt further training would be beneficial with one nurse highlighting this would help with *“difficult conversations” (H3)*, stating that it can feel *“uncomfortable…telling someone they’re metastatic” (H3)* when they feel inadequately prepared for these situations. Agreeing with this sentiment, another commented that *“(we’re) not given any guidance… on how to navigate these conversations” (H2)*. There was a particular desire for additional communication training around *“breaking bad news” (H11)* expressed by all HCP groups. One AHP reflected on their experiences:

The situation where a patient is given bad news is something that happens a lot, and that scenario is repeated and repeated. So, actually, some basic information or tips on how to respond, how to communicate with patients at this time (would be useful), because managing those conversations can be very difficult. – H2

There was also a view that training should be offered regularly so HCPs can frequently update their skills to ensure they are able to continue providing high-quality care. One nurse voiced their surprise that training is not *“updated on a regular basis” (H9)*:

Interestingly, from my personal experience, the level 2 psychology training, you receive that training, then that’s it, off you go. And you have advanced communication training, then off you go. There’s no ongoing, kind of professional development. – H9.

The opinion that continual training would be helpful was also expressed by an AHP so that skills and knowledge were kept up to date with new information:

I think in general it would be good to have regular updates and not only training once in a while… because I think this all evolves and what could be relevant now might not be relevant in six months. – H10

However, the need for regular training was juxtaposed with the challenge of *“finding the time” (H8)*, which affected their ability to engage with additional training. This was highlighted by one nurse:

It was on a day when we have 5 clinics, so there was no time that we could do it… there was no way we could kind of fit it into our work schedule. – H3

An AHP further suggested that having limited resources also influenced the extent to which training is offered, specifically to new employees:

A lot of staff will come here, train for a year and then disappear, so any training that we put into them with regards to psychosocial care would disappear… so there’s always a reluctance to invest too much training into people until we know they’re going to be here for quite a long time. – H5

One HCP felt that additional training would not benefit them due to their position as an oncologist however, linking this with how they perceived their role in relation to additional support provision:

No, I wouldn’t need further training. My role is to treat patients with cancer. I would need somebody to offer that support alongside my role… the support should be offered by the team around me. – H11

### Recognition of HCPs’ own Psychosocial Needs

HCPs acknowledged that their own emotional needs in providing psychosocial care are closely intertwined with patients’ wellbeing. The necessity of recognising the needs of entire cancer care team was identified by one AHP:

I think it’s the people at the forefront, like the chemotherapy nurses or the cancer nurse specialists who are seen as needing (additional support), and a lot of people on the periphery are forgotten…but everybody who picks up the phone to a cancer patient requires that kind of support. – H2

All HCP groups highlighted that they *“absorb a lot of emotional output” (H7),* which can negatively affect their own wellbeing. One oncologist reflected on this challenge:

I think we are all guilty of just, kind of, ploughing on and you know, just getting on with the task and not sort of you know, stopping, reflecting, taking time to think, you know, ‘I need to ask for help’. I think we are generally really bad at doing that, which is probably why our burnout rate is so high. – H8

This view was also expressed by one of the nurses, who commented that *“we’ll all burn out if we don’t get time to reflect” (H3)*. The benefit of *“talking to other colleagues… to decompress or debrief” (H6)* following emotionally loaded situations was also mentioned. A similar sentiment was shared by a member of the nursing team who compared informal reflection with colleagues to clinical supervision, which was previously available to facilitate reflection:

Informally as a team we’ve been quite good at kind of reflecting back at team meetings and doing our own kind of clinical supervision, but certainly none of us are psychologists. – H3

Another nurse reiterated the value they attributed to clinical supervision in fostering their own and other HCPs’ wellbeing. This was considered an essential tool through which they could develop both professionally and personally:

I personally think it’s quite like clinical supervision. Is it a taboo that you do clinical supervision because you’re not coping? No, it’s there because it helps you cope, and it gives you the resilience and resources and tools to help you cope in the future. – H7

HCPs highlighted how attending to their own wellbeing is crucial to providing patients with *“the care they deserve” (H10)*:

We’re not psychologists. We’re not able to provide ourselves, I think, with the psychological support for coping with people that we may have known for 10 years and who suddenly are deteriorating. And I think that’s really important to have, because we if we’re not right, then we can't help our patients. – H2

## Discussion

This qualitative study has explored what patients and HCPs at a single cancer centre in the UK consider to be the core priorities in relation to psychosocial cancer care. Eight themes were identified, six of which were cross-cutting and two which were specific to HCPs. The findings of this study illustrate the multifaceted nature of psychosocial wellbeing, supporting extant literature indicating that a cancer diagnosis can impact individuals’ lives in numerous and different ways.^61^ As such, psychosocial support should be considered an indispensable aspect of the cancer care pathway.^62^

Our study takes a novel approach by qualitatively exploring, combining and contrasting the views of patients and healthcare providers (HCPs) on psychosocial cancer care. Notably, the shared core priorities between these groups indicate a strong alignment in perspectives. These findings are particularly valuable for those seeking to understand the diverse factors influencing patient and HCP wellbeing throughout the care pathway. Additionally, policymakers may find the results of this study useful in developing further educational resources to enhance existing support for patients and HCPs.

The importance of making sure each patient is cared for in a way that supports their unique needs was also highlighted, supporting findings from previous work indicating that patient-centred care improves satisfaction,^63^ wellbeing^50^ and health outcomes.^64^ This is further reflected by the global guidelines emphasising a need to provide appropriate needs-based support to each patient.^25,65^

Although many opinions shared by patients and HCPs were congruent, there were some discrepancies in the data. Differences particularly arose within the patient sample in terms of their support preferences and core priorities. These factors included and went beyond just physical health and emotional wellbeing, encompassing other aspects such as being provided with information to guide them through their cancer journey and practical aspects of recovery. The data also reflected that patients do not equally require the same quantity or style of psychosocial support. Therefore, a stepped-care approach whereby more resources are allocated to those who require more support^66^ could be adopted to promote more efficient use of these.^67^

Awareness of additional resources, even when these were not perceived as being needed, was also valued by patients; this awareness empowers patients to take an active role in making decisions about and acting on their evolving care needs throughout the cancer care pathway.^68^ There was also discordance between HCPs and patients regarding the challenges perceived by HCPs regarding restricted time and resources for psychosocial support provision; whilst HCPs vocalised concerns about how this could be detrimental to patients, patients themselves did not feel their care was compromised by these constraints.

Although some areas where care could be improved were identified, these were relatively few, and patients’ views were generally positive. However this could be due to selection bias among the sample; patients with more positive experiences may have been more likely to be approached by the hospital team to participate in the study.^69^ Moreover, existing literature has noted that patients can be reluctant or find it difficult to provide negative feedback about care in order to preserve the rapport they have established with their hospital team^70^ or due to concerns this will negatively impact their care.^41^ Thus, these factors need consideration for their potential to bias the data.

The mode and time of psychosocial care delivery also warrants consideration. Practicalities affecting access to psychosocial support were identified, which have been acknowledged as a barrier in previous work.^31,32^ Online support groups can help to overcome these barriers and supplement care^71^ as they can be accessed more easily and are widely available.^72^ These however introduce their own challenges, with uptake of and adherence to these varying depending on patient factors such as gender and age.^73^

The role of care coordination was also clear. When care was well-coordinated patients felt greater reassurance, particularly when it translated across the entire healthcare team. This reflects previous qualitative work which has identified that when healthcare teams work collaboratively, this fosters greater psychosocial care provision.^33^ Greater continuity of care has also been associated with lower care needs over a 12-month follow-up period^74^ illustrating its lasting impact on living with and beyond cancer. Conversely, poor communication between primary care HCPs and cancer specialists is a key challenge in care delivery and can result in difficulties accessing or sharing information.^75^ Lack of care coordination has already been cited as a significant factor in negative care experiences^76^ and was acknowledged as *“never great” (P8)* by patients in this study.

Patient-HCP communication was another common theme from the data. These interactions are an integral aspect of patient-centred care^77^ contributing to health-related quality of life^78^ and increased satisfaction with care.^79^ The UK National Institute for Clinical Excellence (NICE) guidelines have established the fundamental role of communication in relation to patient outcomes.^80^ The National Cancer Institute in America similarly consider this an essential part of HCPs role in caring for patients.^81^

Further, the impact of HCPs’ own needs in providing psychosocial care merits consideration. Clinical supervision, whereby individuals can reflect on the impact of their work in a protected space with a more experienced practitioner^82^ was frequently mentioned. This can be valuable for HCPs who are faced with death, dying and other situations that are emotionally loaded.^83^ Cancer health professionals are also thought to be particularly at risk of burnout due to their heightened emotional stress,^84^ which can adversely affect patient satisfaction and quality and safety of care.^40^ Providing HCPs with appropriate resources and support to minimise this risk has accordingly been identified as a major priority in the UK,^85^ USA^86^ and Australia.^87^

Coordination of care across different hospitals is recognised as playing a part in supporting wellbeing. The UK NICE guidelines^80^ and the ASCO recommendations, for example, include optimising care coordination for best professional practice and patient outcomes.^88^ Some patients reported a high level of coordination in their care whilst others found this not to be the case. There were comparisons made regarding the better organised internal coordination within the cancer centre and poorer coordination between sites and Trusts. This mirrors previous work which identified an association between lack of coordination and worse care experiences.^89^ Meanwhile greater care coordination improves patient outcomes by better guiding patients through the healthcare system and enhancing cooperation between medical teams and healthcare providers.^61^

As has been established in previous research, time and additional resource constraints were identified as key barriers in providing psychosocial care to patients.^33,35,36^ Lack of care provider knowledge and consequent signposting behaviour^90^ negatively impacts the utilisation of additional support resources by cancer patients. It has also been acknowledged in other research that lack of referral to support resources after treatment completion detrimentally affects quality of life within patients’ survivorship period.^14^ Lack of referral behaviour is exacerbated by HCPs’ requiring ongoing training on psychosocial support^91^ combined with their restricted capacity to remain informed about resources^92^ impeding signposting behaviour. There are also barriers to HCPs accessing resources to develop their own skills and knowledge. These include electronic resources not being easily accessible^93^ or requiring substantial financial and time investments,^94^ difficulties with integration into practice^95^ and perception of psychosocial care in relation to their professional role.^36^

Another finding from this study is that HCPs actively expressed a need and desire for advanced training in communication skills, particularly in delivering bad news and discussing disease progression. HCPs spoke about the importance they attach to continued education and the need for frequent learning opportunities to better support care provision, noting a gap in current training in terms of providing psychological support to patients. This is important for shared decision-making, ensuring patients are informed about their condition, available options and implications of their choices.^96^ The benefits of further training have been well-established in previous research, such as enhanced team collaboration,^97^ improved communications skills,^98^ self-efficacy and performance.^99^

As with existing research,^38^ there was a particular discussion around continued education and additional communication training. Our findings particularly reflect that of previous literature which has reported on the challenges HCPs may face when communicating with patients, especially those with metastatic disease.^39^ A consensus meeting of European oncology communication experts identified communication training as essential and suggest that this should be mandatory for doctors and nurses.^100^ This reduces the likelihood of providing information to patients without also offering psychosocial support where required,^98^ improves HCPs’ attitudes towards psychosocial care and bolsters confidence in recognising psychological concerns.^36^

### Strengths and Limitations

There were several strengths of the current study. Interview topic guides were developed through a rapid review and refined using focus groups, ensuring rigour in this. One author (ZC) coded the entire dataset and created the thematic framework initially and these were verified by a second author experienced in qualitative methodology and oncology research (JA) for accuracy and completeness. Using a qualitative methodology enabled rich data collection and provided insights that may not have been captured quantitatively. The perspectives of patients and HCPs were equally considered in this research and is a significant strength of this work, highlighting how many of the main priorities for patients and HCPs overlapped. A variety of HCPs were included in the sample, thus representing the experiences of the wider cancer care team rather than a specific subsample of this.

There are limitations to this work, however. The sample size included more patients than HCP and both were recruited from the same cancer centre. However, our study had information power due to its focused research question, specific sample and rich data, ensuring sufficient depth and relevance to address the study aims effectively.^57^ There is the possibility of selection bias in the sample; patients with positive experiences may have been more likely to be invited by hospital staff to participate or be more willing to contribute to this research. There was an uneven gender split in both participant groups, which is attributable to the convenience sampling method selected to meet the target sample size and facilitate timely completion of data collection. The greater proportion of male patients we interviewed could also be viewed as a strength of our study as they tend to be under-represented in studies investigating psychosocial support. Details about participant age were not collected, which could have provided insight regarding further variation in psychosocial care priorities.

### Implications and Recommendations for Practice

As psychosocial priorities differ among patients, a stepped-care approach is essential to ensure patients’ unique needs are met. We suggest that the format, mode and timing of psychosocial care delivery should be carefully considered to ensure that this is equitably accessible by the broad range of people affected by cancer. Furthermore, we recommend encouraging continued development for HCPs to supplement their existing skills and empower them in psychosocial care delivery; this is particularly relevant for communication skills training, whereby high-quality communication with patients fosters greater shared decision-making and patient involvement in care. However, implementation into their workflow needs to be considered. It should also be borne in mind that HCPs can be psychologically affected by the nature of their role and may also require additional support to minimise burnout risk and enable continued high-quality care provision.

Future research could extend this across multiple geographic locations and cancer diagnoses to increase the diversity of the sample. Future research could also explore HCPs’ expectations regarding their role in providing psychosocial support to cancer survivors and their relatives. The views of informal carers were unrepresented due to lack of uptake by this population, so further work could explore their priorities. Strategies to improve the reach and accessibility of psychosocial resources such that these can be implemented into current care practices could also be evaluated.

## Conclusion

Our study confirmed that psychosocial support is an integral component of patient care encompassing several different factors. There is no single element that we established as ‘*what matters most’*, rather, priorities differed among and between patients and HCPs as well as throughout the entire cancer pathway. Participants reported that when care is individualised to match patients’ needs and preferences, their wellbeing and satisfaction with care improved. Additional support, when required, should be provided in ways that enable greater reach to those who wish to utilise these. We also found that it is also important to remain mindful of HCPs’ own wellbeing given its role in their ability to provide high-quality care. Further, adopting a stepped-care approach is recommended for optimal care provision as practitioners will better be able to personalise care and target psychosocial support to those who need it most. This will in turn will improve patient outcomes and job satisfaction for HCPs.

## Supplemental Material

Supplemental Material - A Single-Site Qualitative Study Exploring What Cancer Patients and Health Care Professionals Consider to be the Core Priorities in Relation to Psychosocial Cancer Care Supplemental Material for A Single-Site Qualitative Study Exploring What Cancer Patients and Health Care Professionals Consider to be the Core Priorities in Relation to Psychosocial Cancer Care by Zoe Clothier, Jenny Harris, Agnieszka Kehinde, Victoria Mumford, Kate Upshon, Clare Williamson, Rachel MacArthur, Rachel Stevenson, Sophie Otter, May Teoh, and Jo Armes in Cancer Control
