# Supplementary material for: A Single-Site Qualitative Study Exploring What Cancer Patients and Health Care Professionals Consider to be the Core Priorities in Relation to Psychosocial Cancer Care
Source: Cancer Control. 2025 Aug 13;32:10732748251356320. doi: 10.1177/10732748251356320 (PMC12351091; doi:10.1177/10732748251356320)
Supplement: Supplemental Material - A Single-Site Qualitative Study Exploring What Cancer Patients and Health Care Professionals Consider to be the Core Priorities in Relation to Psychosocial Cancer Care [file sj-pdf-1-ccx-10.1177_10732748251356320.pdf]

Qualitative paper highlights 20.12.24

- Psychosocial care is pivotal to cancer patients' wellbeing and positively influences their perceptions of health, especially when it aligns with their individual needs and preferences.
- Cross-cutting themes highlighted the importance of holistic approaches addressing the multifaceted needs of patients.
- Supporting HCP wellbeing and providing appropriate training and skill development may enhance the quality of psychosocial care delivery.
- A stepped-care approach is recommended for optimal care provision as HCP will better be able to personalise care and target psychosocial support to those who need it most.

Supplementary Document 1 Interview Topic Guides

Topic guide – staff

[Hello/how are you etc].

Thank you so much for agreeing to take part in this interview, on behalf of the COCARE research team I'd like to express our sincere gratitude. Broadly, the aim with this project is to co-design the psycho-oncology services so that they provide consistent, high quality psychosocial care for patients and to also enhance staff wellbeing. These interviews, in particular, want to explore 'what matters most' for psychosocial cancer care provision, and so your honest thoughts and comments will be vital in helping us achieve our aims.

We will be presenting anonymised quotes from these interviews at the co-design events (which are part of the next phase of the project). We will ensure that you personally cannot be linked to these quotes in any way.

I will first need to record you providing your verbal consent to take part in this interview, and will stop the recording. I will then resume the recording so that your consent is kept separate from any other information you provide during the interview. I will begin the interview by asking for a brief overview of your job role, and then move on to explore the psychosocial cancer care experiences. (Ask if they want cameras on or off).

STOP RECORDING AFTER CONSENT, RESUME FOR INTERVIEW CONTENT.

……………………………………………………………………………………………………..

- Confirm what their role is, which tumour team, if they work across others, i.e. for oncologists, radiologists), how long have been in their current role (e.g. as CNS etc) and working in team at Surrey

So we are interested in the ‘pinch points’ you and your team might experience in supporting patients/relatives with their emotional or psychological needs, so we can help co-design resources and training to better support this. With all these questions it would be helpful if you could reflect/focus on the more psychosocial needs or aspects of care although of course they are inter-related with the physical etc

1. Tell me about some of the **most challenging aspects for your team in providing psychological care or support** to your patients [Probe: relatives/families of patients too]

2. What are the **main reasons why [key things they identified] are challenging?** *

- What resources, skills or training to you think your team needs to be able to better manage these challenges?
- Practical challenges as well as psychological? Training?
- *How do you make a decision about the best referral pathway for emotional support? What referral pathways do you use in different situations?*

[If not already covered]: How do you feel about the **support and information you have access to with when signposting to psychological support?** Are there any areas where you feel you or others could have more support or training?

3. If you were to think of a **new colleague or less experienced person what do you think they need to know about how to support patients** psychological care?*
    - *Training? Skills? Mentoring? [e.g., if senior doctor might want them to think about more junior doctors]*

4. Thinking about psychological support**, what do you think your team does well?** * (probe why and how achieve this)

5. **And where do you think your team could improve** or it does less well?* (If lots, maybe get them to think of the main areas)

6. Without disclosing any confidentiality, I wondered if you could tell me an example of a situation where you felt the emotional aspects of care for a patient or relative could have been managed better?*
    - What made that quite so challenging? *

7. To finish off, we will be asking a similar question to patients and their relatives, but we also want to get staff views - **what do you think matters most to patients and their relatives** in terms of their psychological care/support? *
   - Do you think they value psychological wellbeing as much as physical?
   - What do you think would be the **main issues that patients or relatives would want staff to know** about their experiences of psychological support/care?
   - What do you think they would be positive about? What do you think they will say about what could be improved? What do they prioritise?

8. Finally **thinking about you, what (if anything) matters most to you** in providing psychological or wellbeing support to your patients?*

Finish off/close; anything else to reflect on & thank for time

Topic guide – patient/relatives/ carers

[Hello/how are you].

Thank you so much for agreeing to take part in this interview, on behalf of the COCARE research team I'd like to express our sincere gratitude.

The aim with this project is to update the psycho-oncology services so that they provide consistent, high quality psychosocial care that is consistent with areas that are highlighted as being 'what matters most' in receiving psychosocial cancer care, and so your honest thoughts and comments will be vital in helping us achieve our aims.

We will be presenting anonymised quotes from these interviews at the co-design events (which are part of the next phase of the project). We will ensure that you personally cannot be linked to these quotes in any way.

I will first need to record you providing your verbal consent to take part in this interview, after which I'll stop the recording. I'll then resume the recording so that your consent is kept separate from any other information you provide during the interview. To start, I will ask you for a brief overview of your cancer journey, and then move on to explore your experiences with psychosocial cancer care. (Ask if they want cameras on or off).

STOP RECORDING AFTER CONSENT, RESUME FOR INTERVIEW CONTENT.

………………………………………………………………………………………………………

1. To start with, please could you tell me a little bit about **yourself and your cancer journey**?

- Tumour site/status
- Initial diagnosis and cancer treatments
- Where they are on the journey now

So, we are interested in your experiences of receiving emotional or psychological support, so we can help co-design resources and training to better support this. With all these questions it would be helpful if you could reflect/focus on the more psychosocial needs or aspects of your care, although of course they are inter-related with the physical.

2. What **care experiences did you find the most challenging** when going through your diagnosis and treatment? (If relevant, And how about later in your journey?)

Probes:
- What impact did these challenges have on your life?
- Did you speak to anyone about these challenges?
- What and/or who helped you through these challenges?

3. What has your **experience of emotional support from health care staff** during your cancer journey been like? Were there any particularly positive or negative experiences?

Probes:

- Did any staff ask you about your emotional wellbeing or how you were doing? How did they respond?
- Did you feel “cared” for by staff? (Why? Why not?)
- Did they signpost support services? If yes, which?

4. How did you find most of the **information to support you** (e.g., about treatments, practical/financial or emotional support)? Was there **any type of information that you thought was missing?** Is there something you wished you’d known more about or had been told?

Probes:

- What did you find helpful?
- What do you think would have helped you at the time?
- Did it feel like the staff members had shared information? Did your care feel coordinated?

5. Looking back, **what has mattered most in relation your emotional wellbeing** throughout your cancer journey?

6. And thinking of where you are now, **what matters most for your emotional wellbeing now?** How could this best be supported? Out of all these experiences, what do you feel were the most challenging for you and why?

7. Thinking about your cancer journey- if you think about all the things make up our wellbeing – such as physically being able to do things, whether that’s at work or home life; being able to socialise or get out and about, for some people this includes spiritual or religious wellbeing too, **how important would you say psychological and emotional wellbeing is in relation to these other aspects**?

8. Thinking back about your whole experience **what are the main things you would want staff to know** about the experience and **how they could improve** emotional support for people with cancer? Thinking back to the early days of your diagnosis and treatment, is there anything you would want to know which you didn’t know then?

9. Is there **anything else** you would like to reflect on or tell us about?

[Thank for their time]

Supplementary table 1: Additional quotes for each theme

**Personalised support:**	
Patients	*I'm not one to go around talking about it with everyone, I'd rather just speak to the people I need to speak to. – P1*<br><br>*If I'm just not sure, I can call her and she just. Yeah, just puts my worries to rest. Yeah, she's just honest and gives me facts as I need. – P1*<br><br>*The nurses were very good at, they realised when I was struggling with sleep and was exhausted, and they would do their best to get me a room on my own for a bit. – P2*<br><br>*I was referred to lots of support services. I didn't take anything up because, to be honest, I didn't feel I needed to. – P3*<br><br>*To be honest, I haven't felt I needed that support. I'm pretty self-sufficient, I'm pretty assertive. I do my research. - P3*<br><br>*I'm quite practical about things, realistic about things. I kind of get on with things, process it in my own mind and get through it. – P4*<br><br>*It's just nice to have a bit of talk and, of course, I don't want to be... I'm not very deep on things anyway, but I expressed myself and say what I think about things… but the answer about psychological support is, I don't need it. – P7*<br><br>*I'd say I'm fairly hard-skinned in that sense. I feel like the emotional support that they offer, and the support given emotionally at [the cancer centre] outweighed my expectations by a considerable amount. – P8*<br><br>*I'm the sort of person that prefers to know what's happening and what's going on. But as I say, everybody's journey is different and it's a very unknown situation. – P9*<br><br>*It helps me to talk about it, but as you say, everybody's different. So I've got a very dark sense of humour so we, especially with my brother and sister, we have a laugh about things. – P9*<br><br>*I have found that unless you do ask specific questions, they don't necessarily give you the give you answers or give you information because they just don't know. – P9*

	The lung cancer nurses, they put me in touch with all the right people, but without overloading me. – P9
HCPs	*We, from psychosocial aspect, just try to keep people's spirits up, try to just be friendly, try to make it a place where they'll… not necessarily enjoy coming, but certainly that they don't dread coming to. – H5*<br><br>*There's all sorts of reasonable adjustments that we can go ahead with. The patient that I specifically referred to actually attended for an hour before even going for their consent for radiotherapy, because they are so anxious they wanted to see exactly what they were getting themselves into before they even had the conversation about agreeing to have it. We're happy to make those reasonable adjustments as well. – H5*<br><br>*A lot of people say, 'oh look, I'll just get on with the treatment, I'll be fine. I don't need to talk to anybody'. And then you get their relatives ringing up. They are you ringing you up 9 months later, saying 'they haven't coped very well. Can you please help them?' But each time you speak to them, they say 'I'm fine', and it's about looking beyond that. – H7*
**Accessing and awareness of support available:**	
Patients	*I can't genuinely, I can't remember, I think it was self-referral. I remember getting a pamphlet from oncology, and I think my partner phoned them and started the process. – P2*<br><br>*There was so much going on in my life outside of my diagnosis, really awful things. I did speak to my nurse about that and she said, 'well, let's go and see if you, do you want to go to the [information and support centre]? Do you want some counselling? – P4*<br><br>*I'm straight in there, straight away saw my oncologist and put on the treatment straight away, so I'm very grateful for the speed of it. It was all very efficient. – P7*<br><br>*Realistically, getting to [the cancer centre] is a, it's quite a pain at the best of times. And yeah, from our standpoint it, it simply wouldn't be practical. –P8*<br><br>*I genuinely think, yeah, we would have enjoyed it. It was a few sort of massages and things like that, all of those sorts of things. Well I think, yeah, my partner and I would have really enjoyed and utilised if it was closer to us. – P8*

	I just, you know, they're accessible, always. My nurse is always accessible, I can speak to her or email her, and she always comes back. – P10
HCPs	*I think because the [information and support centre] is you know, within the [cancer centre]. If the patient is in clinic the nurse would then take the patient to the [information and support centre] and then they can have a discussion about what available resources they can have, or supportive measures they can have. – H1*<br><br>*I tend to direct people to [the information and support centre] because then I know that if they go there, they will meet somebody at the desk who will be able to talk to them and will be able to give them all of the information. – H2*<br><br>*I don't think there's enough joined-up writing as far as signposting people, and I think it would support us to be able to say, 'okay, so this patient is struggling with this. This is a resource that they can access'… then you can point them in that direction. – H2*<br><br>*They really wanted to kind of meet each other and it's not something we've been able to facilitate. We wanted to but haven't had time to… whether that be we just go, there's this group, here's the details and that's self-regulated by them and we're not involved… I think it would be quite good just so they could go to the pub, have a chat, go for a walk… do something that wasn't hospital based, that isn't reminding them of their diagnosis as well. – H3*<br><br>*We've got the [information and support] centre here, which is fantastic and really supportive and the counselling that they've given to our patients, they've all fed back has been wonderful and they've really needed that. – H3*<br><br>*So they have to self-refer, which is quite difficult because often… I think that's really hard if you're in a really low place to, even though it's online to just, put your details in and to refer yourself. – H3*<br><br>*We do have occasions where we have patients where it's not, the [information and support centre] probably isn't appropriate, it's another level up and it's how you access that and when, when it's appropriate to do that. – H4*<br><br>*Yeah, definitely more familiarity with what could be offered would be useful. – H5*<br><br>*Uh, so yeah, in an ideal world there could be more done in the immediate day-to-day treatment base, but again that would just require far more staffing. – H5*<br><br>*I think sometimes, while self-referral is a good idea, sometimes it's difficult for people to kind of take that first step to access some psychosocial elements to their care... I think some patients would like it to be done for them in a way so that the onus is taken off them, and actually their details are passed on… and then somebody contacts them rather than having to do that themselves. – H6*

Patient-HCP communication:	
Patients	*It would be nicer to actually see people than have phone appointments… a lot of them say they'll have me up on the screen, I forget what they call it, video calling, I think. But they don't work. I mean, I've had the computer on, sitting waiting for it and the phone rings and it should come up on the computer but doesn't. Then they say, 'we'll carry on with the phone.' But that's technology letting us down there, not the actual people. – P5*<br><br>*It's better when you get to see your consultant, because he knows you inside out and he knows exactly what is happening. – P9*<br><br>*I do get to meet up with my consultant every two weeks, it's good like that. – P9*<br><br>*The young lady with the consultant was there and she talked to me, and then I said, 'well, is there anything at all that we can do?', thinking to myself that there's no hope really because it had gone to the brain. But 'oh yes', she said, 'of course there's something we can do'. – P10*<br><br>*I get all the information and every time they speak to me, or if I have anything face-to-face, that's all followed up with a letter to my doctor and copied to me, so I am kept well-informed. – P10*
HCPs	*I think it would sometimes… just the language we use or just having the confidence to just, actually have confidence in sharing you know that kind of information perhaps? Yeah, I think I think I feel comfortable now having done it for a few years, to be able to have those conversations. But it took me a long time to feel comfortable, and it still feels uncomfortable telling someone. You know, like when you tell someone they're metastatic and they say, 'am I going to die?' – H3*<br><br>*Sometimes I think when you have a conversation with patients, they don't always quite realise that that is what they need as well, the patients. It's sort of, it's sort of have to delve a bit deeper and then and then it's almost like a light bulb for them, 'Ohh yeah actually I needed a bit more support with this'. – H4*<br><br>*You do miss those, you know, subtle signs that somebody's not doing as well and whether that's a physically not doing as well or mentally not doing as well and you just miss those kind of subtle signs on the telephone. – H6*<br><br>*One of the things I do feel that perhaps changed things is when everything went to more remote communications. So we do a lot more telephone reviews and that sort of thing. And I feel you don't always pick up on the body language side of things that you do when you're face to face with patients. – H7*<br><br>*Sometimes for certain cases you, you don't really appreciate, you know, their psychological struggles until you've got to know them a bit more and you know, you've had the chance to sort of, yeah, see them a couple of times and talk to them – H8*<br><br>*I think if you can review a patient face-to-face, or review a patient on the phone, you can get a different kind of impression about*

	what psychosocial needs they have. – H9.
**Coordination of care:**	
Patients	*I'd say it's all been really smooth. Sometimes I think that the diagnostics, so if I've been for a scan, some people who deliver the results to me I don't always find as clear or explain to the point where I think, 'right, this is where we're going next'… but I've always had the skin team to fall back to and confirm that it's all good. – P1*<br><br>*No, it's really coordinated, actually. Yeah. Like, but again, the linchpin being the nurses, you know, they really are the linchpins of it all, massively so. – P2*<br><br>*That's the downside, that they don't, different departments don't seem to refer you on, you know. You've got to sort of fight to get referred on… you just feel like you are going around in circles, the various departments don't link together. – P5*<br><br>*I've just found them great and consistent and there. And yeah, I can't see how they can improve this, this fantastic service I've had and very grateful. – P7*<br><br>*No, there isn't much in the way of communication between [the cancer centre] and [local hospital]. That's why I say the [cancer centre] have been very good, they've sort of been proactive about it, whereas if I've gone to speak to the specialist nurse at [local hospital] I'm almost updating them entirely on the care itself. – P8*<br><br>*And when I go for treatment, they're all very good. They all know what's happening. If medication has been prescribed for me from the doctor, because I had to go on steroids, that's there for me always… There's never been any hitch in anything, it's amazing. – P10*
HCPs	*You know, sometimes people fall through the net, and they come to us and they're like, 'we didn't know who to talk to and we didn't understand about anything', so at our initial consultation we do address all of those things. And if you know, in the early stages, they've got somebody they can contact if they're not sure what any of it means… we're trying to work at that and make that a bit better. – H4*<br><br>*I think our team are quite tight and we can get all that information, but it's the ones that are getting referred back to their referring hospitals that perhaps, that information doesn't get fed back afterwards. – H4*
**Time and resource constraints:**	

Patients	*"I mean, what I would say is and you know one thing would be maybe a dedicated e-mail to somebody. It's a difficult one, isn't it? Because like, I would speak to the CNS nurses. But they're so busy. Sometimes I feel really bad saying, 'hey, can you chase that PET scan for me?'" – P2*<br><br>*"What can you say? Everybody's up to their eyes in things, aren't they? I mean, as I said, I work for a local authority, and I know how bad things are at there." – P4*<br><br>*"Sometimes I've seen a junior doctor on their own, or the registrar on their own. In other sort of experiences with hospitals, the consultant has come around with his team. I guess they're all so busy that they don't necessarily have the time to split the team up and join every meeting." – P9*<br><br>*You know they are so busy. So for him to meet every two weeks, I'm guessing with most of his cancer patients and I have no idea how many my consultant has but there's always a load in the in the waiting room and and it takes up a lot of his time – P9*
HCPs	*I think within the time limit of the clinic, we probably don't provide as much psychosocial support as we should… especially those who are coming in for the first time and especially for the first consultation where they learn about their disease. – H1*<br><br>*Sometimes healthcare professionals we're too rushed. We maybe don't signpost people, but they are going to need to be given those sign postings. – H2*<br><br>*I think that's probably the biggest support that we can, we can give people, is actually giving them the time. Which is a huge luxury. – H2*<br><br>*…with our work, you know, with our workloads. I kind of outlined at the beginning how broad ranging our job role is. I'm full time. I have a colleague who's full time... It can be difficult, if we're busy. – H3*<br><br>*I personally don't feel like I am giving enough. You know, I feel that some of the patients are missing out on a bit of that care because of, yeah, the lack of time. – H4*<br><br>*Usually the main challenges are when we're just so busy, that we haven't got that time to offer. So that is probably number one. – H4*<br><br>*Outside of that day-to-day provision, there's very little that we have any time to provide. So we have a specific review team who do speak to the patients. They do go into a little bit more detail about psychosocial aspects with them. But ultimately again, it's a signposting avenue to try and help patients. – H5*<br><br>*I think patients actually do realise time is really stretched. The reason I say it is I had a recent patient who she's out with blue, she was she mentioned in clinic that she had been phoning the Macmillan kind of hotline. I found that quite curious because you know,*

	normally they're very much encouraged to contact their nurses about anything and to discuss their concerns. I did um query that bit with her and she said that, you know, the she felt the nurses were so busy and she just needed someone to talk to. – H8<br><br>*Again, I have no capacity to be involved with that, yeah. We've got 30 patients in three hours. You can't do it. – H11*
**'What matters most' in psychosocial cancer care**	
Patients	*It's those little things that actually become really psychologically important when you're in there. Access to fresh air, fresh fruit, you know? – P2*<br><br>*Feeling that I was being cured. Which I did feel, after each and every radiotherapy session. – P3*<br><br>*Honestly, the flexibility of the teams, that they… It's not just a matter of, you know, booking an online appointment. It can be, 'oh yeah, we'll slot you in here, don't worry, we'll sort it.' So yes, they are, they've moved a lot around to accommodate me, which has been absolutely brilliant, and it's meant that my disorganisation at this point is nullified to an extent. – P8*<br><br>*Truthfulness, if I'm honest. You know, although it's a difficult picture, they did inform me about everything. And also hope, you know, that there's hope. Which is good because I didn't think I had very long and here I am. – P10*
HCPs	*I think what would matter more, most to patients, is to know that there is someone they can get in touch with when they need to. Someone who isn't going to talk to them about, you know, survival or side effects, but will deal with them emotionally or psychologically. – H1*<br><br>*I think we really pride ourselves first and foremost of getting in early, to support people as early as possible, so that they're not spending ages worrying about certain aspects of their care. – H4*<br><br>*I think for everyone, the main thing we can do to support them if that friendliness, to let them know that they're being looked after. – H5*<br><br>*I do remember reading something recently about where staff thought it was really important that patients knew was it from the HCPC? Saying staff thought it would be really important that patients know they're professional or that they're qualified and that they're trained… But patients were like, 'we don't actually care about that. You're here in the uniform. We trust that you know what you are doing. We just want you to be nice and talk to us and listen to us'. – H5*<br><br>*I can't necessarily solve all of their problems, but I think providing them with the time and the kind of open communication is what I value the most and I think unfortunately don't always have the luxury of having all of that time, but… but I hope that when people come into my clinic room, that they feel that, you know I am. You know, my time is theirs and I'm focused on them, and I'm not distracted by other things, and I'll give them the time that they need. – H6*

	I'd like to think that, you know, that patients feel supported in what can be a very, very difficult time for them, and I would like to think that the patients are, you know, they feel that they're getting the best care possible. And that the team looking after them know what they are doing and that they care. You know, that they are open, willing to help. – H8<br><br>*One always wants to leave just a little bit of hope, even in the end-of-life situation. You know, often it's not possible, but if you can't provide hope in terms of longevity, then you can provide hope in terms of, you know… you won't suffer, you know you'll be looked after. So, I think that's what matters most, that patients have something to hold onto. – H11*
**The necessity for education and training in psychosocial care:**	
HCPs	*I know it might not be available in every cancer centre, but at least, I think, maybe some courses, some webinars, lectures on this particular topic would also be very useful. So either someone we can have a discussion with… or you know, online training, at least. – H1*<br><br>*Now I've been doing it a long time. I've done level 2 psychology training - only up to level 2, but I so I've done some psychology training to support me, and advanced communication skills, but I've got members of my team coming in who haven't had that experience, and I think it's it we need to be able to recognise this, and I don't think we always do – H2*<br><br>*I've had slanging matches in my clinics that if I was 25, I would have found much, well, me at 25. I'm not suggesting every 25-year-old, me at 25 would have been very difficult to manage. But because I was 20 years older or even 30 years older, I was I was able to navigate those conversations. – H2*<br><br>*So we did like level 2 psychological training. I personally didn't find it helpful at all, because the things that they were talking about, I was dealing with stuff that was far more, do you know what I mean? It was far more advanced than that. – H3*<br><br>*I think, I don't know, you know, kind of what's out there, but... this stuff we had before, I felt was pitched too low. I felt if there was something that was pitched a bit higher, you know… I think I'd find that really, really helpful. – H3*<br><br>*Yeah, I think that (additional training) is something that would be helpful. Especially as we've got some new staff coming in, that that would be something that would be great for them to go on. And for all you know, the rest of the team to sort of refresh ourselves with. – H4*<br><br>*There's always lots and lots of training that staff could be given, but it would almost be a never-ending series of training if you tried to capture absolutely everything. So I'm not sure what the best scenario is, but there's definitely scope for more training. – H5*<br><br>*I think I suppose there's a piece of personal development, but also the more kind of team development and service development. There's kind of separate, really. I mean as a team and a service, there's always so much more we can do, but it's just finding the*

	time, the right people in the right job to be able to do that. – H8<br><br>*I think for me maybe it will be more beneficial to have some sort of a degree of update, because all our clinical practices are changing all the time, isn't it? – H9*<br><br>*I think more training, relevant training… it kind of should be updated on regular basis. – H9*<br><br>*I do believe that having a more embedded um psychological support in our training would definitely help. – H10*
**Recognition of HCPs own psychosocial needs:**	
HCPs	*Having the support of my colleagues, knowing I can talk afterward about how something went. I think sometimes when you know you're seeing someone to give them horrible news. You wake up in the morning thinking, 'oh, I'm gonna have to really change someone's life today, this is horrible.' But for me, yeah, support, kind of a debrief. Sometimes it goes better than you expected, and actually you don't need that much, but sometimes it doesn't. – H3*<br><br>*For me, my way of destressing is always exercise, or doing something nice… you know, do something nice, you need to kind of shake it off and remind myself that as awful as it is, I don't need to take on board their emotions. – H3*<br><br>*I've got a good colleague who I, you know, if I'm worried about something I speak to gain his advice. So yeah, I think, I think talking to other talking to other colleagues to kind of decompress or debrief. – H6*<br><br>*As nurses and doctors, we all put up with, I guess the emotional burden and things like that because it is part and parcel of our job. But actually it's, it's okay to say, 'I'm not coping.' It's okay to have that lunch break, it's okay to actually go home on time. – H7*<br><br>*I personally think you should reach out to people if needed, because you can't do your job properly if you're not feeling good yourself. – H7*<br><br>*So my CNS colleagues used to tell me, I don't know if, they've probably stopped doing it now, but they used to have these session which they call supervision sessions with a psychologist. And I thought that was a really good idea, and why didn't we have that as consultants? Because I thought, you know, that would benefit us. – H8*<br><br>*What I mean is we need reminders as we are also humans and we do forget, we have everything going on as well apart from professionally. So I do believe that having a more embedded psychological support would definitely help with that, and tools that would allow us also to become more self-aware of our own emotions and our own needs cause if we are aware of ours, we will be more I would I think that we will be more able to understand and to identify and others. – H10*<br><br>*I continue to say it's fundamental to focus on our own mental health and wellbeing and only by doing so we will be able to reach our patients and their families and provide the care they deserve. – H10*
